# Why does it matter to individuate the senses: A Brentanian approach

**DOI:** 10.1111/ejop.12735

**Published:** 2021-10-26

**Authors:** Guillaume Fréchette

**Affiliations:** ^1^ Department of Philosophy University of Geneva Geneve Switzerland

## Abstract

How do we individuate the senses, what exactly do we do when we do so, and why does it matter? In the following article, I propose a general answer to these related questions based on Franz Brentano's views on the senses. After a short survey of various answers offered in the recent literature on the senses, I distinguish between two major ways of answering this question, causally and descriptively, arguing that only answers giving priority to description and to the classification involved in it are on the right track for a general answer to the related questions. In the second part of the article, I argue that Brentano's descriptive psychology is an attractive candidate for such an answer. His descriptive psychology provides a plausible account of the classification involved in description, in particular regarding the classification of sensory qualities. I close the article by briefly explaining how Brentano spells out the priority of descriptive answers over causal ones.

## 1 THE BASIC QUESTION

What makes smelling the Gruyère cheese and tasting the Gruyère cheese two different experiences? Both experiences are obviously sensory, and they both involve the same object. However, we are naturally inclined to say that these are not only different experiences but also different types of experiences. What then makes these experiences instances of different *types*, *classes*, *groups*, or *kinds* of sensory experiences? To put the question more generally, how do we individuate the senses, what exactly do we do when we do so, and how does it matter? I will call this Question Q.

In the following article, I first propose a short survey of the answers to Question Q offered in the recent literature on the senses (Section 2). I distinguish between two major ways of answering this question, causally and descriptively. I argue that the answers that give priority to description over causal explanations are fundamentally on the right track, depending on how they spell out this priority.

These answers are on the right track, I maintain, because any answer to Question Q must start by saying something about the basic experiential fact that when we individuate the senses, we classify our experience and because classification is a descriptive task.

The basic experiential fact is twofold: First, it seems that we individuate our experiences on a primitive basis in terms of the qualitative aspects of the content of our sensory experiences. This kind of individuation, or classification, simply seems embedded in our experience. It may well be perfectible, correct, or incorrect, but the essential point about this primitive, or basic, classification, as I will argue, is that we naturally, or pre‐reflectively, consider it to be reliable. This makes it more fundamental than the causal explanation and also allows us to identify the phenomenon that will ultimately be the object of a causal explanation.

Second, we also individuate our experiences in terms of their modalities, for example, as cases of seeing and hearing, and this seems quite obvious, in cases of both veridical and non‐veridical perceptions. Our sensory experience is subject to errors, disturbances, and irregularities of various sorts, such as illusions; hallucinations; cases in which errors or discrepancies are produced by damages to the brain, the nerves, or the sensory organs; or cases of conditions such as synesthesia, some of them being subjectively indistinguishable from cases of so‐called veridical perception. Even in such cases, we do individuate our sensory experiences as cases of seeing or hearing, for example. Subjective indistinguishability is a fact of our experience that even disjunctivists accept.^1^ Excluding cases of subjective indistinguishability, and of non‐veridical cases in general, as irrelevant for answering to Question Q amounts to miss what is perhaps central to sensory experience in general.^2^

In the second part of the article (Sections 3, 5), I argue that Franz Brentano's descriptive psychology offers a plausible account of these two aspects of the abovementioned basic experiential fact, which makes it an attractive candidate for the kind of account of the individuation of the senses which, as suggested, are on the right track. I argue that Brentano's descriptive psychology provides a plausible account of the basic classification mentioned above (Section 3). I show how this account is used in his classification of sensory qualities (Section 4), and I propose a Brentanian account of the two aspects of the basic experiential fact regarding Question Q (Section 5). I conclude the article by briefly explaining how Brentano spells out the priority of descriptive answers over causal ones. His account is not only on the right track; it is more comprehensive than other accounts on the right track (Section 6).

## 2 TWO ANSWERS AND THE BASIC EXPERIENTIAL FACT ABOUT THE INDIVIDUATION OF THE SENSES

I suggested that there are two main kinds of answers to Question Q. The first kind of answer, the causal answers, follows more or less the organic route: We individuate the senses by considering what seem to be the origins of sensory experience, for instance, the sense organ that makes a specific experience possible: Visual experiences are made possible by the eyes, auditory experiences by the ears, and so on. Along the same lines but in a more refined way, another kind of answer suggests that we should consider the kind of signal or energy received by the perceptual system: Following this idea, it is the difference between signals or energies that determinates the difference between the experiences. Causal answers have their modern origins in Johannes Müller's law of specific nerve energies (Müller, 1840, 1843): The law states that all sensations are the conduction of a state of our nerves to consciousness and implies that sensory modalities should be individuated by specific nerve energies. It was developed further by Müller's student Hermann von Helmholtz (for instance, Helmholtz, 1867, 1924–25) and made its way in neurobiological accounts of the senses up to the present day.

The second kind of answer to Question Q, the descriptive answers, suggest, roughly, that we individuate the senses by considering, in various ways, what is accessible to the perceiver in an experience. According to this general view, I would individuate my tasting the Gruyère as a gustatory experience on the basis of what is accessible to me in the experience itself. There are, of course, different ways to spell out what “accessible to me in the experience itself” means: It can be spelled out in terms of a representational feature (e.g., the red color of what I see), a phenomenal character (e.g., what it is like for me to see red), a special property of the perceived quality (color for vision, pitch, and volume for sound), or other such accessible features. There may be different conditions in which such a feature can individuate a sensory modality, but at bottom, an account along the lines of the second kind of answer states that we must first consider what is accessible in our experience in order to build classes or types of sensory experiences. These classes are then considered the sensory modalities or the senses.^3^

There seems to be an important discrepancy between these two kinds of answers to Question Q: On the one hand, it seems quite reasonable to consider the signal, the source, or, generally speaking, the stimulus as decisive in determining the senses. Signals can act upon receptors: When these receptors are activated in a normally functioning system, we usually have an experience, say vision. Such an answer to Question Q provides a causal explanation: We have two different kinds of experiences *because* two different signals operate on different receptors. While this answer may be satisfactory from a causal point of view, it leaves out of the account what I call the basic experiential fact about the individuation of the senses, namely that we *do* subsume sensory experiences under different classes, even without any proper knowledge of the causal explanation and without a complete knowledge of all the properties of the things we thereby subsume under such classes. By leaving out the basic experiential fact, the first kind of answer indirectly suggests that accounting for it would contribute nothing in answering Question Q.

On the other hand, descriptive answers to Q, such as “well, the experiences just feel differently” or references to a special property, such as “tasting the Gruyère is individuated by the salty quality, and smelling the Gruyère is individuated by the pungent quality of its odor,” seem to proceed the other way around: They take the basic experiential fact mentioned above as the central element in answering Q, but they seem to leave no room for the causal explanation of the differences: The causal origins of our experiencing the Gruyère as a case of tasting seem, then, to be a different problem.

On the face of it, it seems difficult to provide an answer to Q that would do equal justice to the various intuitions spelled out in the descriptive and causal answers; hence, there is the impression of a gap. Recent literature on the individuation of the senses reinforces this impression. Macpherson (2011b, p. 3), for instance, suggests that traditional philosophical views about the senses, or “folk views,” as she calls them—which match, to some extent, with what I call the descriptive answers to Q—are potentially challenged by the amount of new information provided by the neurosciences, or the sciences in general, in what I deem causal answers to Q:

We are receiving a huge influx of new information from the sciences that challenges some traditional philosophical views about the senses. This information needs to be incorporated into our view of the senses and perception. Can we do this while retaining our preexisting concepts of the senses and of perception, or do we need to revise our concepts? (Macpherson, 2011a, p. 3).

Her suggestion is that descriptive answers should ideally adapt to causal answers, as they generally do anyway:

The folk already embrace the idea that the number of *actual* senses is a matter to be determined by empirical findings… the folk concept is such that when new empirical evidence of the right kind is brought to light, that which is taken to fall under the concept of the senses can easily be enlarged without changing or revising the concept (Macpherson, 2011a, p. 22).

Obviously, this way of mapping the relationship between descriptive and causal answers suggests that the latter are quite independent from the former. In her view, it seems that descriptive answers are, at best, loose folk preconceptions of the senses that are in need of improvement through empirical findings. Such a view, I suggest, is a case of the primacy‐of‐empirical‐evidence view.

The view comes in different shades. Macpherson's view rejects revisionism: It rejects the idea that we should revise our folk concepts of the senses. However, there are revisionist varieties of the primacy‐of‐empirical‐evidence view, such as that defended by Stoffregen and Bardy (2001), who suggest that the folk concept of the sense should, in fact, be revised because it stands on the false assumption of separate senses, which is, in fact, in contradiction with the actual findings of perception research and also a hinderance to future research on perception. There are also eliminativists among the defenders of the primacy‐of‐empirical‐evidence view. Keeley, for instance, simply discards descriptive answers as “simply nonstarters for a scientific understanding” (Keeley, 2002, p. 6).^4^

Among those opposing the primacy‐of‐empirical‐evidence view, the views defended are not so easily ordered under a corresponding principle. One view that stands out in this respect because it is also often a target of defenders of the primacy‐of‐empirical‐evidence view^5^ is Nudd's account of the senses as our “everyday folk psychological concepts” (Nudds, 2004, p. 42, 2011). In his view, proponents of the primacy‐of‐empirical‐evidence view, such as Keeley, have simply “changed the subject”:

“whatever they are giving an account of, it's not the senses as we commonly understand them” (Nudds, 2004, p. 35).

In other words, descriptive answers to Q have priority because they are the only ones providing an answer to Q; causal answers simply address something other than Q. In contrast to Keeley, Nudds believes not only that our folk concepts of the senses should be taken as a starting point in investigating the senses but also that these concepts are what we mean when we talk about the individuation of the senses—an account considering the receptors in sensory stimulation is an account of something other than the senses.

Our folk concepts, which are part of the descriptive answer, should have priority “because of the explanatory significance being in one rather than another such state has” (Nudds, 2004, p. 43). Here, Nudds spells out priority in terms of explanatory power for action and behavior. To be told that someone sees the vase, rather than merely being told that she perceives it, may well make a difference in explaining the behavior of this person. Classifying the senses as we do in folk psychology thus “gives us an explanation of the distinction between different senses. Its having this significance explains why we distinguish the senses” (Nudds, 2004, p. 45).

The account proposed by Nudds is meant to do justice to the idea that we distinguish between different senses on the basis of the different ways we perceive one and the same object. “It is only because the same objects can be perceived in different ways that it can be informative to know, of a particular perception of an object, which way it was perceived” (Nudds, 2004, p. 46). Because he rejects causal answers given that they account for something other than the senses, and because he believes that the senses are ways of perceiving and these ways are accessible to the perceiver in the experience, Nudds' position clearly belongs among those prioritizing descriptive answers, and for this reason, his account of Q is, I believe, on the right track.

Interestingly, there are other positions attempting to combine both the descriptive and causal answers in a general account of the senses. It is worth mentioning, for instance, the account proposed by Casati, Dokic, and Le Corre. According to them, “no criterion seems to be able to account for our commonsensical intuitions and at the same time to explain some recent scientific data on sensory perception” (Casati, Dokic, & Le Corre, 2014, p. 471). In view of this conclusion, they suggest combining the descriptive and causal answers. The case they argue for is called audition, but it could eventually be applied to other sensory modalities. Sounds, they argue, actually have two modes of presentations: a mechanical mode, thanks to which a sound, for instance, may be discerned by a sensory modality different from hearing (by touch, when you feel in your chest the vibrations of a double bass, for instance), and a qualitative tonal mode, thanks to which only the auditory modality is perceived. It is only by preserving our commonsensical concept of audition that we can still account for the qualitative tonal mode of presentation in audition without reducing it to the mechanical mode of presentation. While they do acknowledge the importance of so‐called folk concepts of the senses, it is not clear whether their combination view implies some sort of priority:

Both conceptual analysis and science are right about the ontology of sound, which by its mechanical nature is in principle available to sensory modalities other than hearing. But, possibly, only hearing can represent certain sounds as having a given tonal quality. Therefore, sounds in the narrow sense do not form a physical natural kind; they are at best an important subjective aspect of our sensory experience that common sense tends to regard as characteristic of hearing (Casati et al., 2014, p. 477).

As an answer to Q, the perspectives suggested by Nudds and Casati and Dokic and Le Corre are likely to be on the right track because they say something substantial about the basic experiential fact regarding Q. Before going further, let me spell out this basic experiential fact and its twofold aspects in more details. First, experiencing something involves some form of classification. When you see the navy blue of the wall, you classify the content of your experience as a case of some species of quality, say blue. To be sure, this classification is assessable for correctness. For whatever reason, I might classify the content of my experience of apple green as a case of blue. However, the fact that classifications may be right or wrong should not mask the fact that the classification itself is part of our experience. Experiencing is classifying. This becomes obvious in various ways: For instance, your actual experience typically connects with other experiences you have had. Thanks to association, contrast, or other grouping strategies that may be involved, one will subsume the content of one's experience under some species of quality, even under some quality‐kind, such as color. This subsuming, or classifying, seems to be a quite a widespread phenomenon among human and nonhuman animals. Take the courtship behavior of the male satin bowerbird, who collects shiny blue objects in a bower in order to attract a female. Obviously, his behavior is successful because he discriminates between blue objects and others in his visual experience; that is, he does classify some of the objects of his experience as blue objects. If this kind of basic classification pervades our experience, not taking it into consideration while answering Q misses the point.

Second, as I suggested above, experiencing as such also involves some form of classification of the sensory modality, of which a particular experience is a case. I do not merely experience the blue vase on my table. I have an experience of the blue vase, which appears to me, evidently, as a case of seeing. This experiential fact is equally at play in cases of both veridical and non‐veridical perceptions, but it becomes particularly noticeable in some cases of illusion. Take the McGurk illusion, for instance. The syllables “ba ba” are spoken while you see the lip movement of someone pronouncing “ga ga.” As a result, so the illusion goes, you will perceive the sound “da da.” This illusion works, I suggest, because we classify, on an elementary basis, our experience of the syllables heard as “da da” as a case of hearing and not as a case of seeing or a case of visual speech perception interfering with auditory perception. Even when we are told about the mechanisms underlying the illusion, we simply cannot be rid of our classification of the syllables heard as “da da” as a case of hearing. Here again, we could assess this classification in terms of correctness, asking ourselves whether the classification picks out sensory contents or sensory modalities in the right way, but again, we should not ignore the fact that classification pervades our experience. We should not ignore the basic experiential fact: We *do* subsume different sensory experiences under different kinds. If Q is also a question about the nature of our experiences, then we simply cannot ignore the basic experiential fact about Q. There is a basic classification pervading our experiences that is essential to our understanding of the senses.

Looking back at the views proposed by Nudds and Casati and Dokic and Le Corre, it seems that they can only partially account or the basic experiential fact. In Nudds's case, the fact is accounted for only to the extent that our classifications are used to explain third‐person behavior. Nudds also presupposes that object perception is primary to our understanding of the senses (Nudds, 2004, p. 46). Consequently, he adds, “it's not clear that the distinction has any first‐personal significance: I don't think it matters to Alice which way she perceives the vase” (Nudds, 2004, p. 46). In contrast to Nudds, as I suggested, the basic experiential fact about Q is twofold: We not only subsume the *content* of our experiences under different quality kinds but also subsume tokens of our experiences under different experience kinds, or senses. In other words, as suggested in my reconstruction of the McGurk illusion, it does matter to Alice which way she perceives the vase.

In the case of Casati, Dokic, and Le Corre, it seems that the view proposed only accounts for our folk classification of the content of our experiences but remains silent on our classification of kinds of experiences, providing, therefore, only a partial explanation for the basic experiential fact about Q. Furthermore, given that they propose a combination of descriptive and causal criteria to answer Q, they must still say something about the nature of this combination. Is Brentano's account then better off?

## 3 BRENTANO AND THE BASIC EXPERIENTIAL FACT

The gap between the two kinds of answers to Q is not only an issue of contemporary debates on the nature of sensory modalities. It was already part of the conflict between empiricism and nativism at the beginnings of scientific psychology, in the middle of the nineteenth century. Philosophers and physiologists such as Helmholtz, for instance, championed a version of the primacy‐of‐empirical‐evidence view: Spatiality, they hold, develops in experience; it is not a quality given in the content of our experience. Therefore, accounting for the perception of space first requires accounting for the genetic conditions under which spatial perception unfolds in experience.^6^ Influenced by the physiologist Ewald Hering, Brentano and his students held the opposite view, that we do perceive space, that spatiality (or extension) is imbued, in perception, with the same dignity as qualities (for instance, colors). They held a view that was labeled “nativism.”

In many respects, the conflict between empiricism and nativism is similar to the gap depicted here between causal and descriptive answers: Helmholtz thought that nativism could not be disproved, because he thought that it was not a theory at all. Empiricism should be preferred, according to him, because “the development of perceptions in experience is to a certain extent demonstrable,” as Boring puts it, and because “there is no need of hypothesizing in addition another ground of perception unless positive evidence can be adduced for it” (Boring, 1929, p. 297), a position quite in line with those described above as examples of the primacy‐of‐empirical‐evidence view.^7^

The relevance of descriptive answers, in contrast to the primacy‐of‐empirical‐evidence view, was already identified in Franz Brentano's *Psychology from the Empirical Standpoint* in 1874 and was systematically addressed in his later lectures on descriptive psychology between 1887 and 1891.^8^ This does not mean however that Brentano rejects causal answers to Q: On the contrary, he is convinced of the necessity of what he calls “genetic psychology” for any psychological investigations; furthermore, he believes with Müller and other physiologists that specific nerve energies play a causal role in the individuation of the senses. However, according to him, it is a mistake to apply causal answers to the realm of the mental without further ado, as if we could provide the same kind of exact measurements for mental phenomena as the ones we provide for the physical realm.^9^ The exactness of our analyses of the mental does not depend on such measurements, but rather on the reliability and appropriateness of our descriptions. It is only on the basis and in the light of such descriptions that causal answers to Q can be provided.^10^

In other terms, Brentano's strategy takes the basic experiential fact about Q seriously, the basic fact that we not only classify the *content* of our experiences but also classify our experiences into kinds of experiences or sensory modalities. This is central to Brentano's descriptive psychology. He begins these lectures by starting with an investigation of the simplest parts of our mental life: sensations.^11^ His starting point is not to ask what *makes* different sensory qualities – say, for instance, visual qualities or cases of a kind of a sensory quality, for example, cases of colors; the starting point is rather to ask, “What do we do when we discriminate between colors?” In his 1893 lectures on phenomenal green for instance, he provides two series of arguments against the physiological accounts of colors defended by Helmholtz and Wundt. After showing the limitations and contradictions of such accounts, he argues that one should follow the “testimony of direct intuition” as provided by experts in color experience, for example, by painters.^12^ Here, what the descriptive psychologist does—as well as the painter as a color theorist, for that matter—is to look for features within the experience and its content that are characteristic of this experience and its content and may occur in a similar way in similar experiences. Doing descriptive psychology, in this account, is first experiencing *tout court*.^13^ However, experiencing always comes with some degree of noticing.^14^ You see that white coffee cup on your desk; you experience some quality. However, you never experience the quality pure and simple. You experience it with a certain degree of intensity, such as a case of light grey or yellowish white or as lighter or darker than the white paper on which it stands. You may not realize, at first glance, that the cup is a yellowish white. You may notice this only after paying more attention. What such a case illustrates, for Brentano, is that your experience comes with some degree of noticing. By noticing the yellowish white, you also group this experience and its content together with other visual experiences of a similar quality. In other words, noticing and classifying are built into the experience itself. This basic kind of classification, Brentano holds, pervades our entire mental life, applying, therefore, not only to the kinds of quality in experience but also to the kinds of sensory experiences themselves. This is the kind of basic classification that I consider essential to the basic experiential fact about Q.

### 3.1 Finding the most distinctive mark—Brentano's classification technique

Let us take a closer look at how Brentano proceeds with such a classification. In fact, he applies it not only to Q but uses it as the basic strategy for descriptive psychology in general, as a way to answer the general question “What is it that we characterize as mental?”, the very first question that one should ask in psychological investigations.^15^ This strategy is deployed as early as in his *Psychology From an Empirical Standpoint*, in 1874. There, he sought to identify the features, characteristics, or marks of the mental in its different forms. We do, for instance, experience our judgings, lovings, and so on as being quite different from the objects of such acts and their qualities. One could say, as Brentano himself suggests, that we experience objects and their qualities as given within a certain spatial configuration, or with a certain extension, while our mental acts, such as judgings, lovings, and believings, seem to lack this feature. However, we need a positive mark. Spatiality, as a phenomenological feature, is a mark of the content of our experiences and not a mark of our experiences as such. In considering the positive marks of our experiences, Brentano identified two: their inner perceivability—the fact that we are always aware of them when we have them—and their intentionality—the fact that these experiences are always about something other than themselves.

Finding the marks is nothing but finding the criteria determining the classification. It is a question of determining how phenomena that are obviously similar from some point of view – for example, cats from a zoological point of view or judgments from a descriptive psychological point of view – belong to a single class. Individuals of a class may be similar in more than one respect: The case of mental phenomena illustrates this well. Brentano seems, however, to believe that some marks are more salient than others. This is suggested by his remark that intentionality is what “best characterizes” (*am Meisten kennzeichnet*) the mental, that is, its most distinctive mark.^16^ Indeed, as he remarks, inner perceivability is something that, in our experience, is not so clearly displayed as what the experience is about. When describing your experience of seeing the blue vase, it is much easier to point at the qualities of the content of your experience than at the experience itself.

The idea that some marks may be more distinctive than others may be spelled out in different ways. Interestingly, John Stuart Mill, who was a source of inspiration for Brentano's conception of classification,^17^ exposed a very similar idea in his *Logic* when describing natural groups in contrast with natural kinds. Natural groups, Mill suggests, are those that “would *most impress the attention* of a spectator who knew all their properties but was not especially interested in any” (Mill, 1843, p. 306, my emphasis).^18^ Brentano and Mill seem to have the same phenomenon in mind. What “most impress the attention” is nothing other than the “most distinctive mark,” what is called salience in contemporary psychology. For the sake of simplicity, I will also use “salience” to refer to the phenomenon that Mill and Brentano have in mind.

Considering the fact that salience plays a role in identifying the mark, it seems that “finding the marks of a phenomenon” in descriptive psychology should be distinguished from “establishing the definition of a concept” in analytic philosophy. They are overlapping to some extent. A mark might be said to correspond to that part of a definition we also call the specific difference. If we define the concept of a cat as the concept of a domesticated feline, then being domesticated is the mark, or the specific difference, of cats. For Brentano, it may seem that finding a mark of the mental proceeds in much the same way as providing a definition for it. Something is mental (*psychisch*) if and only if it is a phenomenon (*genus*) that has intentionality (*differentia specifica*). However, the similarity stops here. While definitions are driven by finding marks that are necessary and sufficient, descriptive psychology, I would suggest, is driven by finding marks that are salient. They may well often turn out to be the same marks, but the procedures used to single them out are different.^19^

### 3.2 Classifying the mental—Some guidelines

I suggested that classifying the mental, for Brentano, involves not only finding the marks for singling out the classes but using the marks that are more salient than others to build the class. Why should a descriptive psychologist proceed in this way? When we compare it with other sorts of classifications, classifying the mental has a relevance of its own because of the nature of its peculiar subject‐matter: the experiences we are having, which seem to appear to us with all their features at once. Classifying the mental, then, crucially involves paying attention. To be sure, biological classifications too crucially involve paying attention. However, there is an obvious difference: Plants and animals do not seem to appear to us with all their features at once. Something more than paying attention is required for their classification: A knowledge of biological processes, gene pools, or the phylogeny of some species is essential to biological classification. To be sure, some knowledge of physiological processes may also influence or even improve our classification of the mental, but because the mental seems to appear to us with all its features at once, even a first‐pass, or primitive, classification of the mental based on the marks of the experience must be reliable at least to a minimal extent.

Accepting this distinction between biological and mental classification means that a basic classification of the mental is not relative to the state of our knowledge. It is not relative, Brentano seems to believe, because we access the mental in inner perception, where everything is at it appears.^20^ At this level, the classification of the mental is not constrained by various observations or new discoveries, as it is in the case of biological classification. While biological classification is constrained by information, discoveries, and also practical and theoretical interests, the classification of the mental is constrained only by our ability to pay attention.^21^

Paying attention is something one can train and improve. Musical training is a good example: An untrained listener will usually not easily notice the individual tones in a C‐chord, while a trained listener will most likely be able, thanks to his training, to single them out in his auditory experience. As a descriptive psychologist, when you classify your mental experiences, for example, your tone experiences, your basic classification depends on your ability to notice.

However, the ability to notice has its limits, what Brentano calls a “threshold of noticeability” (*Merklichkeitsschwelle*).^22^ Beyond this threshold, you must use further tools to perfect your classification. You might use experimentation, as Brentano's student Stumpf did, to show that some auditory qualities are multiple and that they are fused.^23^ Plausibly, however, the fusion of tone qualities is something that lies beyond the threshold of noticeability for many descriptive psychologists: Brentano himself maintained that he could not bring himself to notice the phenomenon through training, and he was not convinced by some of Stumpf's experimental results.^24^

Descriptive psychology is thus a matter of mental classification of our experiences as they are given to us. Roughly, the classification is obtained thanks to our ability to pay attention. This ability is constrained by various natural factors, but these constraints are also relative, at least in part, to our ability to improve our attention through training. In other words, descriptive psychology provides a descriptive answer to Q and begins with the basic experiential fact about Q: Describing our experience is also classifying it. This elementary, or basic, classification may ultimately be proven right or wrong within a given perspective, but it is a basic experiential fact because it appears to us as evident. In the McGurk effect, my experiencing “da da” as a case of hearing appears to me as evident as my experiencing “ba ba” as a case of hearing does in a normal situation. Brentano's entire conception of inner perception, what we often call inner awareness, is founded on this thesis: what appears to us in inner awareness appears to us as evident. Let me say a few things about this conception of evidence (*Evidenz*) and how it relates to what I called the basic classification.

Brentano spells out the phrase “appearing to us as evident” in various ways. *Evidenz* comes, according to him, in two main kinds: so‐called axiomatic evidence, such as the law of non‐contradiction, for instance,^25^ and what “innerly appears to us as evident,” which is the evidence of inner perception. Axiomatic evidence has a negative form: It tells us what cannot be the case for logical or conceptual reasons (there cannot be p and non‐p, there cannot be a color without a particular shade). Only the evidence of inner perception has a positive form, which could be rendered in an indexical sentence such as “this (which I am presently experiencing) is a case of hearing.”

When attempting to spell out what *Evidenz* is, Brentano sometimes seems to suggest that it implies infallibility.^26^ This characterization is somewhat confusing because it suggests that propositions expressing inner experiences are necessarily true, something which Brentano does not say. Rather, the talk of infallibility (*Untrüglichkeit*, *Unfehlbarkeit*) regarding the evidence of inner perception means that, in inner perception, we can only but accept (*anerkennen*) what is given to us, that is, the experience we are presently undergoing.^27^ When hearing a song, I may doubt or be convinced that there is a c‐chord I am presently hearing. In inner perception, I do not have such an alternative: I can only but accept that I have an auditory experience. Nevertheless, the proposition “this (which I am presently undergoing) is an auditory experience” is not necessarily true: It is possible that what I take to be an auditory experience is not auditory at all, like in the McGurk illusion. A better characterization that captures the same idea and yet avoids this talk about infaillibility states that what is given in inner perception is evident because, as we said earlier, everything in inner perception is as it appears.^28^

This, I believe, better captures both the intuition that descriptive psychology is a mental classification of our experiences as they are given to us (the descriptive answer to Q) and the basic experiential fact about Q: the *Evidenz* of inner perception is what grounds the reliability of the description and the basic classification, but the reliability of a classification should not be confused with its appropriateness.

Let me return to the McGurk effect to illustrate my point. In the McGurk effect, you may describe your experiencing the heard syllables “da da” (when the spoken syllables are “ba ba” and the lip movements indicate “ga ga”) as an experiencing of *hearing* “da da.” The description and basic classification are reliable because they simply, or trivially, depict the way you experience the heard syllables “da da.” To be sure, whether it is *appropriate* to classify your experiencing of the heard syllables “da da” as a case of hearing is also a question of classification—and therefore a descriptive question as well—but to answering it, we need to go beyond the inner perception of our own personal case, beyond our actual threshold of noticeability. We need experimentation, or controlled observation, as we mentioned in the last section. Controlled observation, or experimentation, according to Brentano and his students, is just a specific case of experiencing. When looking at the green grass in the garden, I may be able to discern, within my experience, the qualities of blue and yellow. In this case, as in any other case, my classification of the color qualities is trivially reliable—I merely discern the parts of something that constitutes my experience and is given to me in full. However, its appropriateness will be decided based on whether I can successfully generalize the case given to me in inner perception, for instance, whether I control the conditions of the visual experience by reproducing it with other observers or introducing systematic variations.^29^ In other words, the *Evidenz* of inner perception is that which makes our basic classifications reliable, and experimentation is what makes it appropriate, for instance, when these classifications are replicable.^30^

This difference between the basic classification made reliable by inner perception and the controlled classification made appropriate, for instance, by experimentation also explains why many descriptive psychologists, even within Brentano's school itself, all agree on what descriptive psychology means, while they disagree on the classification of mental phenomena. Brentano himself was not always convinced by the tripartition of mental phenomena into the classes of presentings, judgings, and acts of love and hate; Meinong (1902, 1983) introduced the class of assumptions between presentings and judgings and divided the third class into two different classes, as did his (and Brentano's) pupil Christian von Ehrenfels (1887) a few years before Meinong. Husserl himself introduced many other classifications of mental acts and questioned the pertinence of using intentionality as a mark of the mental. What might appear to be fundamental disagreements between these philosophers are simply the consequence of them applying different conditions of appropriateness for a classification, using different strategies to control the classification, being in disagreement over the results of experimentation, or even being in disagreement over the outcome of the analysis regarding some of the key concepts at play. More generally, these disagreements may be the consequences of their different ways of interpreting (*deuten*) what is given in inner perception.

## 4 FINDING THE SALIENT MARK AGAIN—BRENTANO'S CLASSIFICATION OF THE SENSORY

In *Psychology from an Empirical Standpoint*, Brentano's descriptive psychology touched on two main classifications: the distinction between the physical and the mental and the classification of mental phenomena. In various lectures and manuscripts, he further developed this classification, which he sometimes characterizes as an anatomy of the mental.^31^ In the first of three lecture courses on descriptive psychology held between 1887 and 1891, the main concern is the classification of presentations, more precisely sensory presentations.

What are the classes of presentations? Brentano answers this question by first considering what is most fundamental within the class of presentations. Some are sensory presentations, such as the actual presentations of colors and tones, while some are simply based on these presentations, such as the memory of seen color, or the abstract presentation of a man. After identifying sensory presentations as the most fundamental presentations, the next step consists in finding marks for this fundamental class. While the basic questions in 1874's *Psychology* were “What are the marks of the mental?” or “How many classes of the mental are there?”, suggesting a search for a classification criterion for *mental* phenomena, the basic question in 1887 is “What are the marks of the sensory?” or “How many (classes of the) senses are there?”, suggesting a search for a classification criterion for *physical* phenomena.^32^

As a starter, as we mentioned in Section 2, all sensory contents have a quality and a localization. In contrast to empiricism, which holds that we only learn to locate things into space, Brentano holds that localization *is* a descriptive feature of the object sensed, that it is accessible as such in every single sensory experience. We also individuate the content of presentation thanks to its spatial moment (Brentano, 1979, p. 67). As a consequence, we can always differentiate between two contents that are otherwise identical by the different localizations they occupy in the sensory space (*Empfindungsraum*: Brentano, 1979, p. 70), which means that every sensory content fills a location in the sensory space. From this thesis follows Brentano's thesis of impenetrability: “as in the physical space where matter is impenetrable to matter, so is in the sensory space quality impenetrable to quality” (Brentano, 1979, p. 70). In other words, the sensory content of yellow in my visual field occupies some given sensory space, and when it occupies it, then this space cannot be occupied by any other quality.

Brentano is convinced that his thesis of impenetrability is proven by various descriptive psychological observations and that it can be confirmed by experimentation.^33^ In a lecture on phenomenal green, he argues that most painters—who are, in his view, the real experts in the description of chromatic experiences—do discern the blue and the yellow qualities dispatched in various proportions and intensities in their experiences of various shades of green.^34^ In other words, in our experience, there are no such things as a green quality, only various spatial arrangements of blue and yellow qualities. He also presents similar data in the field of audition, supporting the impenetrability thesis.^35^

The impenetrability thesis and the supporting analogy with physical space suggest that localization is to the phenomenal space what extension is to the physical space. This seems to be Brentano's view because he sometimes characterizes space as a *quantitative* mark of the sensory.^36^ If we take the analogy seriously, it suggests that while, in the physical space, matter may occupy a given location in different ways (as a solid, liquid, or gas), in the phenomenal space, quality too may occupy a space in different ways (as a color or a sound). It also suggests that, while there are features shared by everything that lies in the physical space, such as volume, mass, and density, there must also be features that are shared by everything that lies in the phenomenal space. Here, Brentano's analyses lead to three such features, three kinds of qualities or marks that are recurrent in all sensory perceptions: Every quality comes with a certain intensity, brightness, and saturation. Intensity, he holds, is the most distinctive mark, the salient mark of the sensory. I see two reasons for considering intensity to be the most salient mark of the sensory. First, quality, in general, seems more salient than localization. This seems obvious in audition. You simply notice the qualities of the tones (high tone, low tone, and whether they are a chord or not) more than the region from which the sound comes in your auditory space. Second, touching upon an explanation of intensity provided by Brentano,^37^ we find intensity, in the same sense of the term, in all sensory qualities. This is not the case for brightness and saturation. Visual brightness and auditory brightness are only analogue, while visual intensity and auditory intensity are identical.

What is Brentano's concept of intensity, and how do all sensory modalities possess intensity in the same sense? If we divide the phenomenal space into single places or fields, as Brentano suggests us to do, we can see that, in the case of auditory qualities, the fields are not always filled. You do not always hear a sound; sometimes, the auditory fields are empty. In the case of visual perception, in contrast, the fields are always filled. This being more or less filled on the part of the fields of a sensory space is what Brentano calls its intensity. Painters, Brentano's experts in chromatic experiences, are able to discern blue and yellow in phenomenal green because they are able to notice the blue and yellow fields within the sensory space of green, and they can tell, on this basis, that the intensity of blue is lower in phenomenal green than the intensity of blue in phenomenal primary blue. In the first case, only (roughly) half of the fields are filled with blue, in the last case, all the fields are filled with blue. In other words, we might not notice the blue and yellow in the phenomenal green, but we do experience them. If intentionality is said to be the mark of the mental, then intensity is definitely the mark of the sensory.

What about saturation? Saturation, says Brentano, builds upon the intensity and the degree of complexion (*Colorit*) of mixed sensations. It is a direct function, as Eisenmeier (1918) puts it, of the relative intensity and degree of complexion. According to this view, a whitened blue and blackened blue are blue qualities with a lower and higher degree of saturation, which is a function of the proportion of white and black in the respective shades. Brentano argues that we find a feature analogous to saturation in all other sensory modalities.

Finally, brightness is the feature according to which qualities are light or dark (in the case of vision), sweet or sour (in the case of taste), hot or cold (in the case of touch) or high or low (in the case of audition). While the hearing of a tone and the seeing of red may have the same intensity (or intensity in the same sense), they always have brightness in a different (analogous) sense.

Therefore, we have one class of qualities, namely, intensity, that *makes* them qualities in the same sense (i.e., the respect in which they are all species of one kind, the “sensory” kind), and we have one specific difference, namely brightness, that individuates specific qualities. In short, Brentano's descriptive answer to Q may be spelled out in the following way: The mark of the sensory is intensity, and we individuate the classes of the sensory, the senses, in terms of their specific brightness.

## 5 WHAT IS BRENTANO'S INDIVIDUATION STRATEGY?

We may accept that intensity is the most salient mark of the sensory, but how does individuation work in the case of vision, for instance? Some quality *x* is a visual quality, it seems, if *x* displays the brightness of a specific kind. If this really is Brentano's individuation strategy, as it seems to be,^38^ then the senses are individuated by a property specific to the exclusive object of this sense. Brightness, in the visual sense, is a property that only the objects of vision have. Such a strategy reminds us of Aristotle's own method and his distinction between the two kinds of objects of the senses^39^: the proper sensible, that is, “what is special to a single sense” (in Brentano's case, visual brightness), and the common sensible, that is, “what is common to any and all of the senses” (in Brentano's case, intensity). Indeed, according to Aristotle, “colour is the special object of sight, sound of hearing, flavor of taste: Movement, rest, number, figure, magnitude are common to all.”^40^ In this sense, it seems that Brentano defends an Aristotelian content view: There is a special “object” of vision, which is color (i.e., that which has visual brightness); there is a special object of audition, which is sound (i.e., that which has auditory brightness). Many of his remarks suggest such an interpretation: Vision, he suggests, is more fundamental than other senses because vision gives us the paradigmatic, or focal meaning, of brightness.

What also speaks for a content view of the individuation of the senses in Brentano is that the classes of “gustatory brightness” and “olfactive brightness,” or the classes of “temperature brightness” and “pressure brightness,” are not as easily separated from one another as the classes of visual and auditory brightness. To put it differently, the special object of taste has too many qualities in common with the special object of olfaction to individuate different sensory modalities. To put it yet differently, the “special objects” of these modalities are not salient enough to individuate a sense. This is why Brentano classifies them under a third sense, namely, the sense of feel (*Spürsinn*, not to be confused with *Gefühl*), which is a rather large class involving almost all other sensory modalities.^41^

Of course, it is right to say, from a Brentanian standpoint, that we individuate vision by its special object and that we individuate audition by its special object. In this sense, Brentano's account shares with the Aristotelian representationalist account of the senses an essential feature.

But what, then, exactly is Brentano's “special object”? Saying that it is color understood as that which has visual brightness, as we did above, requires further explication. I would suggest understanding color, like intentionality and intensity, as salient marks. If you had a perfect knowledge of all the marks given in vision, color would still be a salient mark, to paraphrase Mill. It is also because of the saliency of the mark that color is taken as the individuating mark, or “special object,” of vision.

There are, however, less salient marks that can be isolated if we pay enough attention, that is, if we exercise our noticing adequately. This is where the less salient mark of visual brightness comes into play. When we individuate seeing, the special object “color” is perhaps the most salient mark, but it is not the only mark. When you see a color, it is the intentionality of your seeing (its intentional object) that emerges as salient. There is usually very little noticing at play to single out a color, but when you pay more attention to your experience, when you innerly perceive your seeing, the Brentanian account suggests, you will notice your seeing because of the visual brightness that characterizes a visual content.

How are the intentionality mark and the inner perceivability mark related to one another in individuating the senses? I discussed the evidence of inner perception in earlier sections to stress the way in which our basic classifications are said to be reliable. Inner perceivability is merely the thesis that *all* our mental acts and, of course, all our sensory presentations are conscious, or innerly perceived. Taken together with the intentionality thesis, this amounts to saying that consciousness and intentionality are coextensive and that there are no unconscious (and non‐intentional) mental acts. There is much to say about the plausibility of this thesis. However, if you have already accepted the second aspect of the basic experiential fact about Q, this thesis should sound plausible to you. The idea is this: When you hear a tone, for instance, there is an object of your act of audition which is the sensory content, but you also are aware of your hearing. Brentano speaks, in this context, of the primary object of the hearing (the tone) and of the secondary object (the act of hearing itself with its correlate). If the thesis is correct, we do individuate our senses in inner perception, but how?

Let me propose what seems to me a plausible construction: We do classify our sensory experiences as cases of hearing and seeing, for example. In basic classification, we order them under certain principles, normally based on what is most salient in experience. Following this basic classification, cases of seeing, Brentano suggests, are cases in which all sensory content is colored. Now, think of visual brightness. Unlike color, brightness is not something you “see” when you look at the white cup of coffee on your desk, but it is clearly involved in your visual experience. You only have to bring a second white cup of coffee into your visual field to see that the white of the first cup is brighter than the white of the second cup. Here, inner perceivability also enters into the individuation of the senses: Brightness is not something that you can see like you see colors. Rather, it is the quality of a quality, something that is there in every visual experience but is never the object of the experience itself, unlike colors or sounds. It is something that accompanies your visual experiences. In other words, you can also individuate your experience of red a as case of seeing because you perceive, “en parergo,” or on the side, the quality of a quality, because you have the inner perception of visual brightness.^42^

Brentano's descriptive answer to Q thus has means of addressing the two aspects of the basic experiential fact about Q:

1. How we individuate the *content* of our experience: through a basic classification in which we look for the most salient mark. Intensity is ultimately revealed to be the mark of sensory content in general. Then, we look for the subclasses of the class of the sensory. This proceeds in two ways: (a) by looking for the most salient mark, that is, the intentional object of each of the subclasses; the “special object” of vision, for instance, is color and (b) by paying attention to innerly perceivable marks (and by training ourselves to detect such marks). In the case of vision, this would be visual brightness. Like intentionality and consciousness, color and visual brightness are coextensive.
2. How we individuate the experiences themselves: Through basic classification, when we do not pay particular attention, we usually individuate these experience kinds by their special objects because, as we said, intentional objects are more salient in our experience than the experience itself. When we pay attention, we notice that the hearing is innerly perceived on the side. This would also be Brentano's account of the McGurk illusion.

Why should this double classification method matter? Why should it matter to say that I experience visual brightness, or seeing, *en parergo*? Is the quality of color not sufficient to individuate vision? It seems to me that there is at least one obvious advantage of using multiple marks in cases in which the quality of the object is not enough or does not play the role it should. There are cases of synesthesia in which, for instance, one sees colors while hearing sounds. The synesthete does hear the sound that is played, but experiences colors that are not there at the same time. If inner perceivability would not matter for the individuation of the senses, it would be difficult to make any sense of the synesthete's description of her own experience. What does it mean to “hear colors” if it is not a category mistake? It means that the synesthete is aware of the sensory modality involved (hearing). She cannot be aware of it simply by the sensibilia (say, the sound as object of hearing). We need something more. We need an account in which we are able to individuate our senses by the kinds of experiencings there are.^43^ This is what my Brentanian account is able to offer.

## 6 COMBINING THE DESCRIPTIVE AND EMPIRICAL ANSWERS, THE BRENTANIAN WAY

Before closing this article, there is one last question to be addressed. Even if we accept that descriptive answers should be prioritized over causal answers in terms of individuating the senses and if there is a plausible Brentanian account based on these descriptive answers, what should such an account do with causal answers? Should it merely follow Nudds's position and discard causal answers as having nothing to do with the senses?

Nudds's position seems to me a nonstarter for a Brentanian account. Brentano and his students were highly critical of similar positions. Along with many other Austrian philosophers and economists, Brentano attacked the Prussian idea that the human sciences would have a different object than the object of the natural sciences.^44^ As a result, descriptive and causal answers should not only be compatible but they should also be complementary, as I stressed at the beginning of Section 3.

How does that work for Brentano? In his 1887 lectures on descriptive psychology, he sketches the situation of descriptive and genetic psychology in the following way:

If it were so easy to do descriptive psychology, the difficulty would have been solved long ago, whereas we rather have to begin from the very start. There are signs of disagreement and divergence of views. Indeed, in important aspects, prevalent views, as we shall see, reveal the most decisive errors. On the other hand, many points that are of the greatest interest have not yet become an object of attention. Everything is rudimentary, unfinished, chaotic. Of course, it is a special obstacle, indeed the greatest one, for more significant advances in genetic psychology—which has to fulfill such challenging tasks (Brentano, forthcoming).

In other words, descriptive answers may seem easy to formulate, but because they pertain to classification problems and there is already disagreement within the descriptive answers themselves, providing a good classification of the mental should come first, before investigating its causes.

As we have seen in this article, descriptive psychology is perfectible. We can improve our descriptive answers, but we can also improve our causal answers. In the case of psychology at the turn of the century, many of the explanations provided had a physiological basis, relying on the laws of association. Brentano was well aware that such a basis of explanation was insufficient. He was convinced that the laws of genetic psychology should have a psychophysical nature.^45^ Although genetic psychology is, as such, perfectible, it would remain even at its highest standard an incomplete or inexact science, Brentano believes, because direct measurements of intensity will never be possible in an exact manner and indirect measurements can only be performed in an inexact way. Despite this fact, these measurements are essential from both descriptive and genetic perspectives. They are essential from a descriptive perspective because there are quantities in the realm of the mental: sensory qualities have extension in space, as we have seen, and intensity and brightness, according to the discussion in the previous section, also imply distances between qualities. Measurements are not the task of descriptive psychology, but it requires measurements for its completion.^46^ To put it differently, as applied specifically to the question Q, both descriptive and causal or genetic answers have the same objects, that is, sensory modalities. They both have intrinsic limitations. In the first case, our description is constrained, for instance, by the threshold of noticeability, while in the second case, we can only obtain approximations of measurements of intensity. In this context, the priority of descriptive answers over causal ones regarding Q (or regarding any analysis of the mental) only has to do with the order of investigation. Description and classification come first. When classes are isolated, we can look for an explanation of the causes. In other terms, descriptive and causal answers should be seen as two ordered moments of an investigation about the senses or, more generally, of every investigation concerning the mental.

I stated above that Nudds and Casati, Dokic and Le Corre were on the right track in terms of accounting for Q. We are now in a better position to see the shortcomings of these two positions: Nudds's account disconnects the descriptive and causal answers to Q. It explains the descriptive significance of Q by our way of explaining behavior and action, and it rejects the significance of first‐personal (or inner) experience in answering Q, which considerably reduces the significance of the individuation of the senses. The account of Casati, Dokic and Le Corre is unsatisfactory for two reasons: Not only do they still have to account for the nature of the combination of descriptive and causal answers, but by suggesting that our commonsensical intuitions are descriptive answers to Q, they undermine the Brentanian idea that we must pay more attention to the experiential data on which we base our commonsensical intuition. It is not a matter of adapting our folk concepts to the scientific data. It is a matter of spelling out, in the most precise way, what our commonsensical intuitions already contain.^47^

## Endnotes

^1^
See, for instance, the *locus classicus* of the position in Hinton (1967).

^2^
Gilchrist (2006, p. 266ff) suggests a similar approach, considering the pattern of errors as the signature of the visual system.

^3^
Descriptive answers, as characterized here, include a wide range of positions and have a long history, dating back at least to Aristotle, and his “proper sensibles” (*De Anima*, Book II, chapter 6), whose position is often considered a representationalist view (see, for instance, Macpherson, 2011a, p. 23ff.; Matthen, 2015). Focusing on the phenomenal character of the experience as a criterion, Grice (1962) too proposes a descriptive answer.

^4^
The point is also made in Keeley (2009), where Keeley connects his position to Müller (1840, 1843).

^5^
See, for instance, Keeley's objections to Nudds in Keeley (2012).

^6^
See, for instance, Helmholtz (1879, 1971).

^7^
In particular, Keeley (2012).

^8^
See Brentano (1874, 1973/2015, 1982, 1995). For a new edition of the materials on the origins of Brentano (1982), see Brentano (forthcoming).

^9^
This is the basic line of his critique of Wundt in *Psychology from an empirical Standpoint*, in particular Brentano (1973/2015, p. 73f.).

^10^
See Fréchette (2020b) for a more detailed discussion of this point.

^11^
This investigation builds the whole 1887 lectures, selections of which have been published in (Brentano, 1982, 1995, pp. 134f.,143f.). The content of sensations is also central to the 1888/89 lectures. A few pages from the introduction of these lectures have been published in Brentano (1982, 1995, pp. 129f.,137f.).

^12^
See Brentano (1979, p. 98). The lectures published in Brentano (1979) were first published by Brentano himself in 1907 as a way of providing a published account of his descriptive psychology. He abandoned the plan of a systematic publication of lectures at about the same time. These points are discussed in the correspondence between Brentano and his student Stumpf (Brentano & Stumpf, 2014).

^13^
See Brentano (1982, 1995, pp. 29f.,32f.).

^14^
Brentano (1982, 1995, pp. 31f.,34f.).

^15^
This is the question the second book of *Psychology from an empirical standpoint* answers, Brentano (1874, 1973/2015, pp. 101ff.,81ff.).

^16^
“That mark (*Merkmal*) which best characterizes mental phenomena is undoubtedly their intentional inexistence” Brentano (1874, 1973/2015, pp. 127, 102). Translation modified.

^17^
On this topic, see Hedwig (1987), Fréchette (2012, 2014), and Dewalque (2018).

^18^
See Magnus (2015) on the distinction between natural groups and natural kinds in Mill.

^19^
This also supports Brentano's suggestion (Brentano, 1895, p. 34) that descriptive psychology aims at providing to the mental what Leibniz's *caracteristica universalis* was meant to provide for language.

^20^
This thesis is formulated in many different ways in the *Psychology from an empirical Standpoint*: sometimes, Brentano explains that psychology is a science, which is exclusively phenomenal (*ausschließlich phänomenale Wissenschaft*); that is, it deals exclusively with phenomena (Brentano, 1874, 1973/2015, pp. 20,14). This however should not be taken as a reinstatement of the Kantian duality between phenomenon and thing in itself: As Brentano puts it, the opposition between phenomenon and “things which really and truly exists” only holds for objects of external perception: “it does not apply in the same way to objects of inner perception: in their case, no one has ever shown that someone who considers these phenomena to be true would thereby become involved in contradictions” (Brentano, 1874, 1973/2015, pp. 13f.,9f.). He stresses the same point in the lectures on descriptive psychology: “to be a phenomenon, something must exist in itself. It is wrong to set phenomena in opposition to what exists in itself” (Brentano, 1982, 1995, pp. 129,137). I thank Denis Seron for drawing my attention to these passages. I do not believe however that they commit Brentano to phenomenalism, as he suggests in Seron (2018).

^21^
This does not mean that some pay better attention than others and, *therefore*, perform their classification correctly. Provided that they both rely on inner perception, a poorly gifted descriptive psychologist and a master would both propose reliable classifications. This also explains the disagreements in descriptive psychology, even within Brentano's school itself. I return to this point below.

^22^
Brentano (1979, p. 132).

^23^
See, for instance, Stumpf (1886, 1890, 2020).

^24^
See Brentano (1979, p. 66ff.). On Brentano and Stumpf on fusion, see Martinelli (2013). In addition to the threshold of noticeability, two other elements constrain this noticing: The narrowness of consciousness, that is, the fact that we are able to pay attention only to some extent, determined by fatigue or other similar factors, and the laws of habitude, that is, we notice better when we perceive the same or a similar phenomenon more regularly. These are not absolute constraints: training and various techniques may help to augment some qualities.

^25^
The list is actually longer; see Fréchette (2019, p. 426) for an exhaustive list.

^26^
See, for instance, Brentano (1874, 1973/2015, pp. 182ff, 139ff.).

^27^
Van der Schaar (2018, p. 232f.) exposes further reasons not to spell out evidence in terms of infallibility in reading Brentano.

^28^
See, for instance, Brentano (1874, 1973/2015, pp. 28f., 19f.). Brentano makes this point at other places as well (Brentano, 1968, pp. 9–10) and also characterizes knowledge by inner perception as “distinct knowledge” or “knowledge of the factual,” in opposition to axiomatic knowledge as knowledge “of something as impossible.”

^29^
This point was forcefully made by Franz Hillebrand—a student of Brentano who attended his descriptive psychology lectures—in his own inaugural lecture at the University of Innsbruck in 1896: “Do we need experimentation for the pure analytic description? Isn't the pure self‐observation enough? The mistake lies here already in the question. Experimentation and observation are not opposites; rather, they relate to one another like the species to its kind. When one experiments, one observes too; it is only that one does not observe the phenomenon as it occurs without one's influence, but rather one induces it arbitrarily and according to a plan” (Hillebrand, 2018, p. 62, my translation).

^30^
Here, I discuss experimentation as one of the constraints the descriptive psychologist will apply to her basic classifications, but there are other constraints that are also a part of the descriptive method, along with experimentation, as further species of the kind “description”: demonstrations of examples and exposition of the consequences for instance—what we would call today conceptual analysis—are mentioned by Brentano as such further constraints. See Fréchette (2019, p. 431) on this point.

^31^
Brentano (1982, 1995, pp. 128, 135).

^32^
Brentano (1982, 1995, pp. 153, 162, 1889, 1902, pp. 14, 13).

^33^
Brentano himself often reports conducting such experiments in his correspondence with Stumpf. See Brentano and Stumpf (2014, pp. 328, 333). He explains his limited contribution to experimentation in Brentano (1895).

^34^
Brentano (1979, p. 8ff.).

^35^
Brentano (1979, p. 93ff.).

^36^
Brentano (1979, p. 67ff.).

^37^
Brentano (1979, p. 82ff.).

^38^
See also Eisenmeier (1918, p. 487ff.).

^39^
On the Aristotelian elements in Brentano's individuation strategy, see Taieb (2020).

^40^
Again, Aristotle, *De Anima*, Bk. II, Chapt. 6.

^41^
Brentano (1979, p. 162ff.).

^42^
This reconstruction is supported by the distinction between the intentional object and correlate, which Brentano discusses in various places, notably in Brentano (2013). The idea was already formulated in Brentano (1874, 1973/2015): When I hear a tone, what I hear is the tone (the object) and not a “heard tone” (the correlate). But when I innerly perceive my hearing (the tone), I am not paying attention to the tone anymore, but to the hearing. And yet, the tone is still present, but in a modified way, namely as “something heard” or as a “heard tone.” This is what Brentano designates as the correlate of an intentional act of hearing. This also allows us to individuate a specific sensory modality in inner perception. I discuss this distinction in Fréchette (2013). See Sauer (2006) and Antonelli (2015) on the distinction between the tone and heard tone in Brentano (1874, 1973/2015).

^43^
Of course, not all models of synesthesia suggest that the synesthete is aware of the sensory modality. Models such as Keeley (2013) suggest that she has two conjoint experiences. A suggestion along the lines of Auvray and Deroy (2015, p. 647), in which the concurrent and the experienced inducer are considered two aspects of the same experience, would make more sense for the Brentanian approach suggested here.

^44^
See, for instance, Hillebrand's critique of Dilthey's distinction between the natural sciences and the human sciences in Hillebrand (1884). On the Brentanian critique of Dilthey, see Huemer (2018, p. 59f) and Fréchette (2020a).

^45^
More on this point in Fréchette (2020b).

^46^
These points on measurements of phenomenal space are made in more detail by Brentano in the 1887 lectures; see Brentano (forthcoming).

^47^
A first version of this paper was presented at the descriptive psychology conference in Salzburg in October 2018. Thanks are due to the participants for their valuable input at various stages of the preparation of this paper—Johannes Brandl, Tim Crane, Arnaud Dewalque, Denis Seron, Charles Siewert, and Hamid Taieb—and also to an anonymous reviewer of this journal. This paper has been written as part of the Austrian FWF P‐27215 (Brentano's descriptive psychology) project and the Swiss SNF project 100012_182858 (*Réalismes: Universaux, relations et états de choses dans les traditions austro‐allemande et médiévale*).
